# Supplementary material for: Evaluating alternative hypotheses to explain the downward trend in the indices of the COVID-19 pandemic death rate
Source: PeerJ. 2021 Apr 20;9:e11150. doi: 10.7717/peerj.11150 (PMC8063871; doi:10.7717/peerj.11150)

**Supplementary information**

Downward trend in the indices of death rate in the Covid-19 pandemic: Evaluating alternative hypotheses

[Sonali Shinde^1^sonalisaccount@gmail.com](mailto:Shinde1sonalisaccount@gmail.com)

PratimaRanade1p[ratima532@gmail.com](mailto:ratima532@gmail.com)

Milind Watve^2*^[milind.watve@gmail.com](mailto:milind.watve@gmail.com)

**S1: The indices used to reflect death rate:**

We use two indices to reflect death rate in this paper. They are needed since cumulative CFR is not sufficiently sensitive to time trend and is disproportionately influenced by the time period with maximum incidence. Therefore we use two indices namely the ratio of daily new deaths and daily new registered cases (ND/NC) and daily new deaths and new recoveries (ND/NR). Being calculated on a daily basis they have sufficient time resolution as compared to CFR and being divided by the current incidence, they represent the time trend more faithfully than the cumulative CFR. The main limitation of these ratios is because of the time lag between the day of diagnosis and the day of death or recovery. In a growing epidemic the number of new cases is likely to have increased during this lag making ND/NC an underestimate of true death rate. But for the same reason ND/NR can be an overestimate of the death rate. Therefore, if there are no other sources of biases, the true death rate can be captured between the two ratios quite reliably. When the rate of transmission (Rt) in the population at a given time is constant, ND/NC is a consistent underestimate and ND/NR a consistent overestimate. However, if and when Rt increases, ND/NC will tend to decrease and ND/NR tend to increase. On the other hand, if Rt is decreasing, ND/NC will tend to increase but ND/NR may decrease. If both the ratios are changing in the same direction, it’s a robust indication of a change in death rate irrespective of Rt.

We demonstrate the behavior of these indices in a simulated epidemic. In a simpler model, the incidence grows exponentially, but the deaths and recovery happen with a time lags t_d_ and t_r_ respectively. The model assumes that the rate of spread of infection is limited by the number of infectious individuals and susceptible individuals are not limiting. Such condition can be assumed at the beginning of the epidemic, until a substantial proportion of the population become immune. A proportion of infected individuals (d) die after a time lag of td or recover after a time lag t_r._ Therefore

NC(t) =R_t_.I(t-1)

ND(t) =d.I(t-t_d_)

NR (t) = NC(t-t_r_) – ND(t_r_-t_d_)

I(t) = I(t-1) + NI(t) – ND(t) – NR(t)

In this model, it can be seen that as long as R and d are constant, both the ratios ND/NC and ND/NR remain constant (fig S1 a). If R increases, ND/NC decreases but ND/NR increases and both stabilize at a new value. If R decreases, both change in the reverse direction (fig S1b). On the other hand if R remains constant but d decreases, both ND/NC and ND/NR decrease (fig S1c).


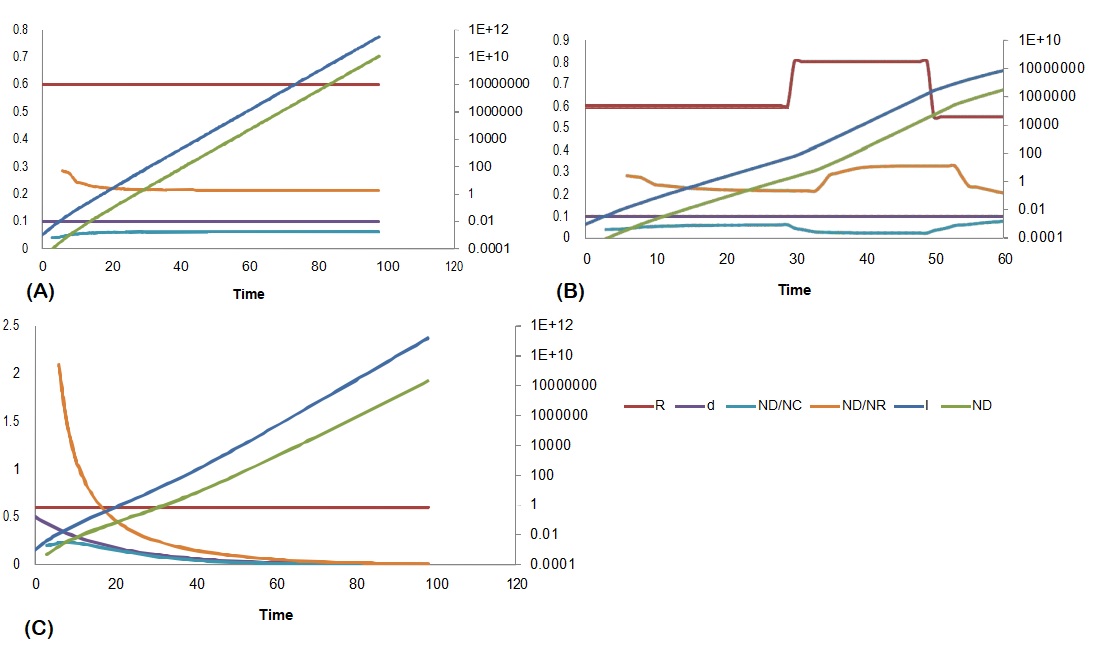

Supplement: Supplemental Information 2 [file peerj-09-11150-s002.docx]
